# The Value of High-Frequency Ultrasound in the Evaluation of Cutaneous Rosai-Dorfman Disease: A Case Series and Literature Review

**DOI:** 10.3390/diagnostics16020242

**Published:** 2026-01-12

**Authors:** Leyan Yang, Minjie Shu, Shuqing Sheng, Haoxuan Liu, Jinyi Deng, Yujing Zhao, Qiao Wang, Lehang Guo

**Affiliations:** 1 Department of Medical Imaging, Shanghai Skin Disease Hospital, Shanghai 200443, China; 2151965@tongji.edu.cn; 2 Department of Medical Ultrasound, Shanghai Skin Disease Hospital, Shanghai 200443, China; 2151717@tongji.edu.cn (S.S.); 2153105@tongji.edu.cn (H.L.); 2250573@tongji.edu.cn (J.D.); gopp1314@hotmail.com (L.G.); 3 Department of Medical Ultrasound, Shanghai Tenth People’s Hospital, Shanghai 200072, China; 4 Hospital for Skin Diseases, Institute of Dermatology, Chinese Academy of Medical Science & Peking Union Medical College, Nanjing 210042, China; smj20190701@163.com; 5 Shanghai Engineering Research Center of Ultrasound Diagnosis and Treatment, Shanghai 200072, China

**Keywords:** cutaneous Rosai-Dorfman disease, high-frequency ultrasound, histiocytosis

## Abstract

**Background and Clinical Significance:** Cutaneous Rosai-Dorfman disease (CRDD) is a rare, benign histiocytic proliferative disorder, accounting for approximately 3% of all Rosai-Dorfman disease (RDD) cases. Currently, the diagnosis of CRDD relies on invasive pathological examination due to the absence of reliable non-invasive alternatives. This case series evaluates the potential utility of high-frequency ultrasound (HFUS) as an adjunctive diagnostic tool for CRDD. **Case Presentation:** We present three CRDD cases, correlating HFUS features with histopathology. All cases showed hypoechoic lesions with varying infiltration depths and morphologies, though no specific diagnostic features were identified. HFUS clearly delineated involvement of the dermal and subcutaneous layers, assessed morphological characteristics like contour regularity and border definition, and evaluated vascularity. This information is crucial for clinical decision-making. HFUS also demonstrated value in therapeutic follow-up. In Case 1, it objectively showed a reduction in lesion size and decreased internal vascularity, providing clear evidence of treatment response. **Conclusions:** Although HFUS cannot independently diagnose CRDD and histopathology remains the gold standard, it serves as a valuable complementary tool. HFUS allows evaluation of deeper tissue structures, infiltration depth, and vascularity. As a non-invasive modality, it is useful for treatment monitoring, therapy guidance, and prognosis assessment. Integrating HFUS into the CRDD workflow enables more comprehensive and precise management.

## 1. Introduction

Rosai-Dorfman disease (RDD), or sinus histiocytosis with massive lymphadenopathy, is a rare benign histiocytic proliferative disorder. It typically presents as painless lymphadenopathy, with cutaneous involvement in about 10% of patients. When confined exclusively to the skin without systemic involvement, it is termed cutaneous Rosai-Dorfman disease (CRDD), which represents approximately 3% of all RDD cases [1]. Although CRDD has a low mortality rate, untreated cases can lead to significant morbidity due to life-threatening visceral involvement [2].

For such rare dermatological conditions, conventional diagnostic methods like dermoscopy often lack specificity, leading to delayed intervention. In contrast, high-frequency ultrasound (HFUS) can visualize deeper architectural characteristics, including infiltration depth, morphological patterns, and vascularization, offering critical supplementary information for evaluating CRDD. However, the systematic application of HFUS in CRDD is poorly documented. This case series presents three CRDD cases, correlating their HFUS features with histopathological findings to demonstrate its value as a diagnostic aid and a tool for guiding clinical management.

## 2. Case Presentation

### 2.1. Case 1

#### 2.1.1. Clinical History

A 41-year-old female presented with an asymptomatic, red plaque on her left cheek. The lesion had been present for 2 years and was of unknown etiology. Physical examination detected no superficial lymphadenopathy.

**Figure 1:**
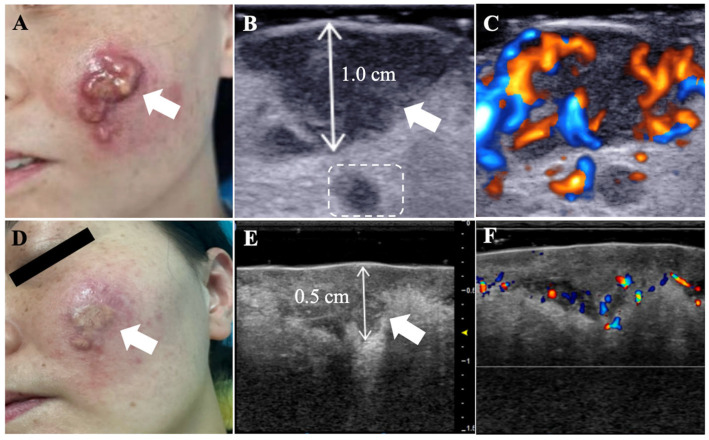
CRDD in a 41-year-old female. (**A**) Appearance of a skin lesion on the left cheek (arrow). (**B**) HFUS (frequency: 15 MHz) of the left cheek shows an irregular, hypoechoic lesion (arrow) with ill-defined borders and heterogeneous internal echogenicity, measuring 3.6 × 1.7 × 1.0 cm. There is a thickened subepidermal low-echogenicity band (SLEB) present at the lesion site. The lesion involves the epidermis, dermis, and subcutaneous tissue, with a deep satellite lesion (dashed box). (**C**) CDFI demonstrates marked intralesional vascularity with multiple thick feeding vessels at its base. (**D**) Appearance of the left cheek after 1 year of treatment (arrow). (**E**) Follow-up HFUS (frequency: 38 MHz) of the left cheek shows an irregular hypoechoic lesion (arrow) with an ill-defined deep border, measuring 1.9 × 1.5 × 0.5 cm. No satellite lesions are observed. (**F**) Follow-up CDFI demonstrates blood flow signals only detected at the bottom of the lesion after treatment.

Physical examination revealed a dark red, irregularly shaped plaque on the left cheek. It was composed of multiple firm, smooth-surfaced nodules with telangiectasia and without ulceration, exudate, vesicles, or purulent discharge (Figure 1A).

#### 2.1.2. Imaging Studies

HFUS (frequency: 15 MHz) revealed an irregular, hypoechoic lesion with ill-defined borders and heterogeneous echogenicity, measuring 3.6 × 1.7 × 1.0 cm. This lesion involved the epidermis, dermis, and subcutaneous tissue, with an associated deep satellite lesion (Figure 1B). Color Doppler flow imaging (CDFI) demonstrated abundant intralesional vascularity (Figure 1C).

#### 2.1.3. Pathological Findings

Histopathological examination of a biopsy specimen revealed no significant epidermal changes. The superficial to mid-dermis showed focal infiltration by lymphocytes, epithelioid cells, plasma cells, and multinucleated giant cells. The immunohistochemical findings included CD4 (+), CD68 (+), S-100 (+), CD1a (−), CD138 (+), PD-1 focally (+). Special staining results were PAS stain (−), acid-fast stain (−). Based on these findings, this lesion was diagnosed as CRDD.

#### 2.1.4. Treatment and Follow-Up

HFUS revealed an irregularly bordered lesion with deep extension and an associated satellite lesion. Given the facial location and the risk of functional impairment, curative resection was considered inappropriate. The patient was therefore started on systemic pharmacotherapy consisting of oral methylprednisolone and methotrexate, supplemented with folic acid and topical emollients.

One year post-treatment, the lesion exhibited significant regression with a flatter surface and lighter pigmentation (Figure 1D). Follow-up HFUS (frequency: 38 MHz) performed one year later showed that the irregular hypoechoic lesion persisted with an ill-defined deep border. There is a thickened SLEB present at the lesion site. However, its dimensions had significantly reduced to 1.9 × 1.5 × 0.5 cm, involving the epidermis, dermis and subcutaneous tissue. The satellite lesion had resolved (Figure 1E), and color Doppler signals were significantly reduced, remaining only at the lesion’s base (Figure 1F).

### 2.2. Case 2

#### 2.2.1. Clinical History

A 42-year-old female presented with an untreated, dark red papule on her right back. The lesion was initially the size of a fava bean and had no identifiable cause. Over the following two months, it enlarged and became occasionally tender.

**Figure 2:**
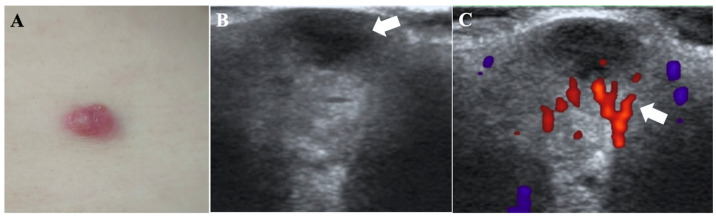
CRDD in a 42-year-old female. (**A**) Appearance of the skin lesion on the right back. (**B**) HFUS (frequency: 22 MHz) of the right back shows an oval, hypoechoic lesion (arrow) with heterogeneous echogenicity, ill-defined borders, and posterior acoustic enhancement, located primarily within the dermis and measuring 1.0 × 0.5 × 0.3 cm. (**C**) CDFI demonstrates absent intralesional vascularity but abundant peripheral flow signals (arrow).

Physical examination revealed a 1.0 × 0.5 cm, dark red, mobile nodule on the right back. It was firm in texture with a smooth surface and no ulceration, vesiculation, or bleeding. Palpation indicated slight infiltration at the base (Figure 2A).

#### 2.2.2. Imaging Studies

HFUS (frequency: 22 MHz) revealed an oval, hypoechoic lesion in the dermis with ill-defined borders and posterior acoustic enhancement, measuring 1.0 × 0.5 × 0.3 cm (Figure 2B). While no significant intralesional blood flow was detected, CDFI revealed abundant peripheral vascularity (Figure 2C).

#### 2.2.3. Pathological Findings

Pathological examination illustrated no significant epidermal hyperplasia. The dermis showed diffuse to focal infiltration by plasma cells, along with interstitial collagen proliferation and sclerosis. The immunohistochemical findings included S-100 (+), CD1a (−), CD20 (+), CD68 (+), PAX5 (+), EBER (−), Bcl-6 (−), Bcl-2 (+). Based on these findings, this lesion was diagnosed as CRDD.

#### 2.2.4. Treatment and Follow-Up

Preoperative HFUS confirmed a well-localized lesion with no significant neurovascular involvement, making complete surgical resection the feasible option. Surgical excision was therefore recommended and performed. The procedure involved complete excision of the lesion under local anesthesia, followed by reconstruction with a random pattern local flap. The procedure was completed without complications. Postoperative instructions included routine wound care, and the surgical site healed without complications.

### 2.3. Case 3

#### 2.3.1. Clinical History

A 13-year-old male presented with a nodule in his left axilla and no identifiable cause. He had undergone surgical excision of a lesion in the same region ten months prior, with postoperative histopathology consistent with CRDD. Multiple new subcutaneous nodules were now palpable adjacent to the surgical scar, raising concern for disease recurrence.

Physical examination revealed an 8.0 cm linear surgical scar in the left axilla. Several subcutaneous nodules, measuring 0.5 to 2.0 cm, were palpable deep to the scar. They were smooth, firm, mobile, and non-tender.

#### 2.3.2. Imaging Studies

**Figure 3:**
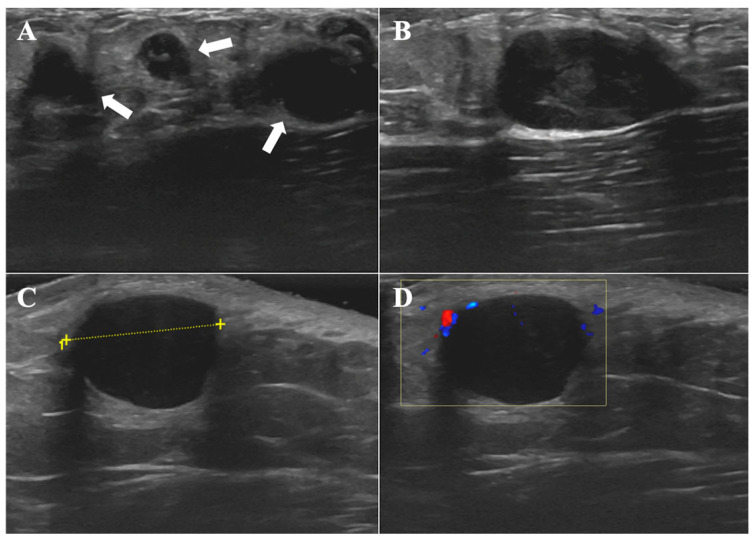
CRDD in a 13-year-old male. (**A**,**B**) HFUS (frequency: 15 MHz) of the left axilla shows multiple, well-defined hypoechoic lesions (arrows) in the left axilla. The largest lesion measures 2.3 × 2.0 × 1.2 cm. (**C**) Follow-up HFUS (frequency: 15 MHz) of the left axilla shows a well-defined, hypoechoic lesion in the left axilla, measuring 1.8 × 1.7 × 1.2 cm. (**D**) Follow-up CDFI demonstrates peripheral vascularity, with no significant internal blood flow.

HFUS (frequency: 15 MHz) revealed multiple well-defined, hypoechoic subcutaneous nodules in the left axilla. The largest of these measured 2.3 × 2.0 × 1.2 cm (Figure 3A,B). No invasion into adjacent vascular or neural structures was identified.

#### 2.3.3. Pathological Findings

The pathological examination demonstrated multinodular histiocytic infiltration accompanied by lymphocytes, plasma cells and neutrophils. The immunohistochemical findings included S-100 (+), CD1a (−), CD68 (+). Based on these collective findings, the lesion was diagnosed as recurrent CRDD.

#### 2.3.4. Treatment and Follow-Up

Preoperative HFUS confirmed a well-defined lesion without neurovascular invasion, supporting the feasibility of complete surgical resection. The patient therefore underwent wide local excision of the recurrent nodule and the surrounding scar tissue. The procedure was uneventful. The postoperative course was unremarkable, with the incision healing well and no signs of bleeding, infection, or other complications.

The patient was re-evaluated 3 months postoperatively. The left axillary incision was well-healed. HFUS (frequency: 15 MHz) revealed a well-defined, hypoechoic lesion in the left axilla, measuring 1.8 × 1.7 × 1.2 cm (Figure 3C). CDFI revealed no intralesional vascularity but demonstrated abundant peripheral blood flow (Figure 3D). These sonographic findings were suggestive of recurrent CRDD.

## 3. Discussion

RDD was first described by Destombes in 1965 and later characterized as “sinus histiocytosis with massive lymphadenopathy” by Rosai and Dorfman in 1969 [3,4]. In 2016, the Histiocyte Society categorised RDD as the “R” Group of histiocytosis, which primarily encompasses RDD and miscellaneous non-cutaneous, non-Langerhans cell histiocytoses. CRDD is classified separately under the “C” Group, comprising cutaneous and mucocutaneous forms of histiocytosis [5].

Sporadic RDD is generally a self-limiting disorder with a favorable prognosis, as spontaneous regression occurs in up to 50% of cases. Treatment options for CRDD include observation, surgical excision, radiotherapy, and systemic therapies such as corticosteroids, sirolimus, chemotherapy, and immunomodulatory agents [6].

This case series characterized the HFUS features in three patients with CRDD to evaluate its clinical utility. HFUS lacks pathognomonic features and is not sufficient for a stand-alone diagnosis of CRDD. However, its ability to delineate anatomical extent, infiltration depth, and vascularity underscores its value in guiding treatment strategies and monitoring the disease course.

Clinically, CRDD primarily manifests as papules and nodules, which may exhibit purplish-brown discolouration, erythema or hyperpigmentation. The lesions show no predilection for specific sites and frequently present in a multifocal or clustered pattern [7], as illustrated in case 3 of our series. Early and accurate diagnosis is challenging because the cutaneous lesions lack distinctive features.

Studies have shown that both dermoscopy and reflectance confocal microscopy (RCM) are useful for diagnosing CRDD. The dermoscopic features of CRDD typically include an orange-red background with pale yellow, millet-like circular areas, surrounded by branched blood vessels, and occasionally white, structureless material. The RCM features of CRDD demonstrate high-refractive round or oval inflammatory cell clusters that have a low-refractive centre and medium-refractive periphery, accompanied by low-refractive branched blood vessels [8,9]. In this manuscript, all three patients presented to the dermatology department with palpable skin masses. Dermoscopy and RCM can provide high-resolution imaging of superficial structures, clearly revealing pigment distribution and vascular morphology within the epidermis and superficial dermis. However, these techniques cannot assess deep tissue infiltration or blood flow distribution. HFUS overcomes these limitations by providing an optimal balance between penetration depth and spatial resolution via frequency adjustment [10,11]. While its spatial resolution is inferior to that of dermoscopy, it enables precise evaluation of lesion depth from the epidermis to the subcutaneous tissue. It clearly delineates infiltration margins and further assesses intralesional blood flow using CDFI. Collectively, our findings illustrate both the diagnostic value and the current limitations of HFUS in the evaluation of CRDD.

### 3.1. Heterogeneity of HFUS Findings and Differential Diagnosis

Only a few studies described CRDD lesions as typically well-defined, hypoechoic, oval or irregular masses with abundant internal vascularity on ultrasound [12,13]. In our series, all three cases demonstrated hypoechoic lesions, which is consistent with prior reports. However, their HFUS features were heterogeneous (Table 1). This heterogeneity underscores the absence of pathognomonic sonographic features for CRDD. Furthermore, the observed features are non-specific and can overlap with those of other benign and malignant cutaneous conditions, complicating the differential diagnosis.

The differential diagnosis for CRDD primarily encompasses other histiocytic and lymphoproliferative disorders [7]. For instance, benign fibrous histiocytoma can present a similar sonographic appearance of a well-defined hypoechoic nodule [14]. Juvenile xanthogranuloma, which is more common in infants, is another consideration [15,16]. Reticulohistiocytoma, may also be considered, as it can be associated with synovial involvement [17]. Lymphoproliferative disorders can be distinguished by their characteristic immunohistochemical and molecular profiles [18]. Although these lesions may have distinctive clinical features, definitive diagnosis relies on histopathological and immunohistochemical examination.

### 3.2. The Role of HFUS in Clinical Decision-Making

Histopathology provides a static, ex vivo assessment. In comparison, HFUS enables a dynamic, real-time, and non-invasive in vivo evaluation, which can delineate the lesion’s complete anatomical extent and its relationship to surrounding structures. In our cases, HFUS clearly delineated the dermal and subcutaneous layers involved, assessed morphological features including contour regularity and border definition, and evaluated the intralesional and peripheral blood supply. This information is pivotal for clinical decision-making. For instance, in case 1, HFUS identification of deep infiltration and a satellite lesion in the facial lesion indicated that conventional surgical intervention might compromise facial function. Therefore, systemic medical therapy was deemed more effective in alleviating the patient’s symptoms. Conversely, the localized and well-defined lesions in Cases 2 and 3 identified by HFUS indicated that they were favorable for curative resection. This contrast demonstrates how HFUS guides personalized treatment planning by optimizing management strategies and avoiding unnecessary invasive procedures when complete resection is not feasible.

### 3.3. The Value of HFUS in Treatment Follow-Up

HFUS demonstrates considerable value in therapeutic follow-up. In Case 1, HFUS objectively documented a reduction in lesion size and decreased internal vascularity during follow-up, providing objective evidence of treatment response. In case 3, it played a crucial role in the postoperative setting by detecting a new lesion, indicating disease recurrence.

### 3.4. The Limitations and Prospects of This Study

One limitation of this study is the use of varying transducer frequencies (15–38 MHz) across different cases and follow-up examinations. However, this approach aligns with clinical practice by tailoring the examination to individual lesions for optimal diagnostic imaging. The primary focus of this study is not the direct comparison of absolute sonographic features between cases, but rather to demonstrate the comprehensive value of HFUS in evaluating infiltration depth, adjacent structures, and temporal changes within individual cases. Moreover, due to its retrospective design and the fact that examinations were conducted based on clinical need, the equipment and parameters for HFUS were not fully standardised across cases.

Furthermore, HFUS demonstrates the potential for real-time biopsy guidance. In cases involving deep, non-palpable, or sonographically heterogeneous lesions, real-time HFUS guidance enables precise needle placement into the most suspicious regions, such as areas exhibiting the most abundant vascularity or the most abnormal echotexture. This precision serves to maximize the diagnostic yield and prevent missed diagnoses resulting from sampling errors or inadequate tissue retrieval. Consequently, we propose that HFUS-guided biopsy be integrated into the CRDD protocol, especially as a standardized procedure for complex or deep-seated lesions, offering an effective complement and refinement to conventional blind biopsy techniques.

In conclusion, although HFUS lacks the specificity to independently diagnose CRDD and histopathology remains the gold standard, it plays a complementary role in the diagnostic workflow. Unlike dermoscopy or RCM, HFUS enables the evaluation of deeper tissue structures, infiltration depth, and vascularity. HFUS thus serves as a valuable, non-invasive tool for monitoring treatment response. It plays a crucial role in guiding treatment planning, enabling longitudinal monitoring, and assessing prognosis. Integrating HFUS into the diagnostic and therapeutic workflow for CRDD facilitates more comprehensive and precise management of this condition.

## 4. Conclusions

In summary, while HFUS features of CRDD are variable and non-specific, precluding definitive diagnosis, it plays a critical role in defining lesion extent, infiltration depth, and vascular distribution. It is a valuable non-invasive tool for monitoring treatment response and disease course, complementing the diagnostic workflow and guiding comprehensive CRDD management.

## Figures and Tables

**Table 1:** Summary of main clinical and radiologic characteristics.

	Case 1	Case 2	Case 3
Age	41	42	13
Sex	female	female	female
Location	left cheek	right back	left axilla
Physical examination	an irregular, dark red plaque with multiple firm, smooth-surfaced nodules	a dark red, firm, freely mobile nodule with a smooth surface	several mobile, firm, subcutaneous nodules with smooth surfaces
HFUS features at presentation			
Regular shape	−	+	+
Well-defined borders	−	−	+
Echogenicity	hypoechoic	hypoechoic	hypoechoic
Involvement layers	epidermis, dermis and subcutaneous tissue	dermis	subcutis
CDFI features at presentation			
Abundant vascularity	+	−	−

## Data Availability

The data presented in this study are available on request from the corresponding author due to availability of images.

## Abbreviations

The following abbreviations are used in this manuscript:

CRDD	Cutaneous Rosai-Dorfman disease
RDD	Rosai-Dorfman disease
HFUS	High-frequency ultrasound
RCM	reflectance confocal microscopy
CDFI	Color Doppler flow imaging
SLEB	Subepidermal low-echogenicity band
